# The Effects of Heart Rhythm Meditation on Vagal Tone and Well-being: A Mixed Methods Research Study

**DOI:** 10.1007/s10484-024-09639-0

**Published:** 2024-04-12

**Authors:** Elizabeth J. Tisdell, Branka Lukic, Ruhi Banerjee, Duanping Liao, Charles Palmer

**Affiliations:** 1 Adult Education Graduate Programs, Division of Health and Professional Studies, Penn State University, Harrisburg, PA USA; 2 Department of Surgery, Penn State College of Medicine, Hershey, USA; 3 Department of Public Health Sciences, Penn State College of Medicine, Hershey, USA; 4 Department of Pediatrics, Penn State College of Medicine, Hershey, USA

**Keywords:** Heart rhythm meditation, Vagal tone, Heart rate variability, Meditation, Respiration, Well-being

## Abstract

Many studies have examined the effects of meditation practice focused on the normal breath on vagal tone with mixed results. Heart Rhythm Meditation (HRM) is a unique meditation form that engages in the deep slow full breath, and puts the focus of attention on the heart. This form of breathing likely stimulates the vagus nerve with greater intensity. The purpose of this study was (a) to examine how the practice of HRM affects vagal activity as measured by heart rate variability (HRV); and (b) to examine how it affects participants’ well-being. 74 participants signed consent agreeing to: (a) take a six-week course to learn the practice of HRM; (b) engage in a daily practice for 10 weeks; (c) have their heart rate variability read through ECG technology and to take two validated well-being instruments at the beginning and end of the 10 weeks; and (d) participate in a focus group interview examining their perceptions of how the practice affected their well-being. 48 participants completed the study. Quantitative findings show the effect of the practice of HRM approached significance for multiple measures of HRV and vagal tone. An increase in well-being scores for those who did the meditation more than 10-minutes per day did meet statistical significance. Qualitative data indicate: (a) the positive effects of HRM on stress and well-being; (b) the development of a more expanded sense of self; and (c) an increased awareness of the interconnection of the body-heart-emotions and HRM’s role in emotion regulation.

## Introduction

Human beings have been using their breath for engaging in yoga or meditation practices since ancient times (particularly in the East), to control their response to stress, to slow their heart rate and to improve wellbeing. These breath and meditation techniques have become far more popular in the West in the last half century, and scientists have been studying the positive effects of meditation and mindfulness with their attention to breath, and their effects on stress reduction and the enhancement of wellbeing (Brown et al., 2021). Some of these scientific studies have been fueled by Jon Kabat-Zinn’s influence in bringing mindfulness, with its focus on the breath and attention to the present moment, to medicine and then studying the effects of mindfulness-based stress reduction (MBSR) on various aspects of health and wellbeing (de Vibe et al., 2017; Kabat-Zinn et al., 1985). De Vibe et al. (2017) conducted a systematic review of randomized control trial studies of MBSR and found overall positive effects on stress reduction and quality of life. While a variety of practices are used in teaching MBSR, one of them is mindfulness meditation which focuses on the normal volume breath.

Many forms of meditation and contemplative practices focus on the breath. Placing attention on the normal volume breath is a common strategy during meditation for focusing concentration. However, more than using breath as a focus of concentration, the rate and depth of breathing also have a profound impact on the parasympathetic nervous system activation which produces effects on heart rate variability and central nervous system plasticity with the clinical effects of improved emotional regulation and enhanced self-awareness (Lehrer et al., 2020a). Gerritsen and Band (2018) argue that more research is needed on the effects of breath itself in contemplative modalities, since many forms of meditation place attention on the breath as a focus of concentration, but only a few focus on the slow deep full breath. The full breath is important because, the vagus nerve is modulated more intensely by the full breath than by the normal spontaneous breath (Sevoz-Couche & Laborde, 2022).

Heart Rhythm Meditation (HRM) is one form of meditation that makes use of the deep, slow, full rhythmic breath. The HRM meditator inhales and exhales for the interval it takes for 6–8 heartbeats that they learn to feel in the chest. Thus, depending on their heart rate, it provides a breathing rate of approximately 3.5–7 breaths per minute (bpm). (Accordingly, those with a faster heart rate would breathe more quickly than those with a slower heart rate.) This form of breathing distinguishes HRM from other meditation techniques. Another key component of HRM includes placing attention on the region of the heart field where emotions are frequently sensed in an effort to enhance emotional awareness and capacity. Hence these cognitive and emotional components of HRM are likely enhanced by the physiological changes produced by the specific breathing practice. While there have been studies that examine the effects of various kinds of meditation, there have been no published studies examining how practicing HRM and its use of the slow, full, rhythmic breath, affects either HRV or overall well-being (as an example of emotion regulation). Hence, the purpose of this study is to determine: (a) how 10 weeks of practice of HRM affects long term sustained changes in vagal activity as measured by heart rate variability (HRV); and (b) how it affects participants’ well-being.

## Key Concepts Related to Study Purpose

There are a number of concepts and studies related to HRV and heart rate variability biofeedback (HRVB) that form a part of the foundation to this study. These relate to the role of the baroreflex and the sinus arrythmia in HRVB, and the concept of neurovisceral integration and emotion regulation along with well-being as it potentially relates to HRM.

### The Baroreflex and the Sinus Arrythmia in HRVB

**Fig. 1:**
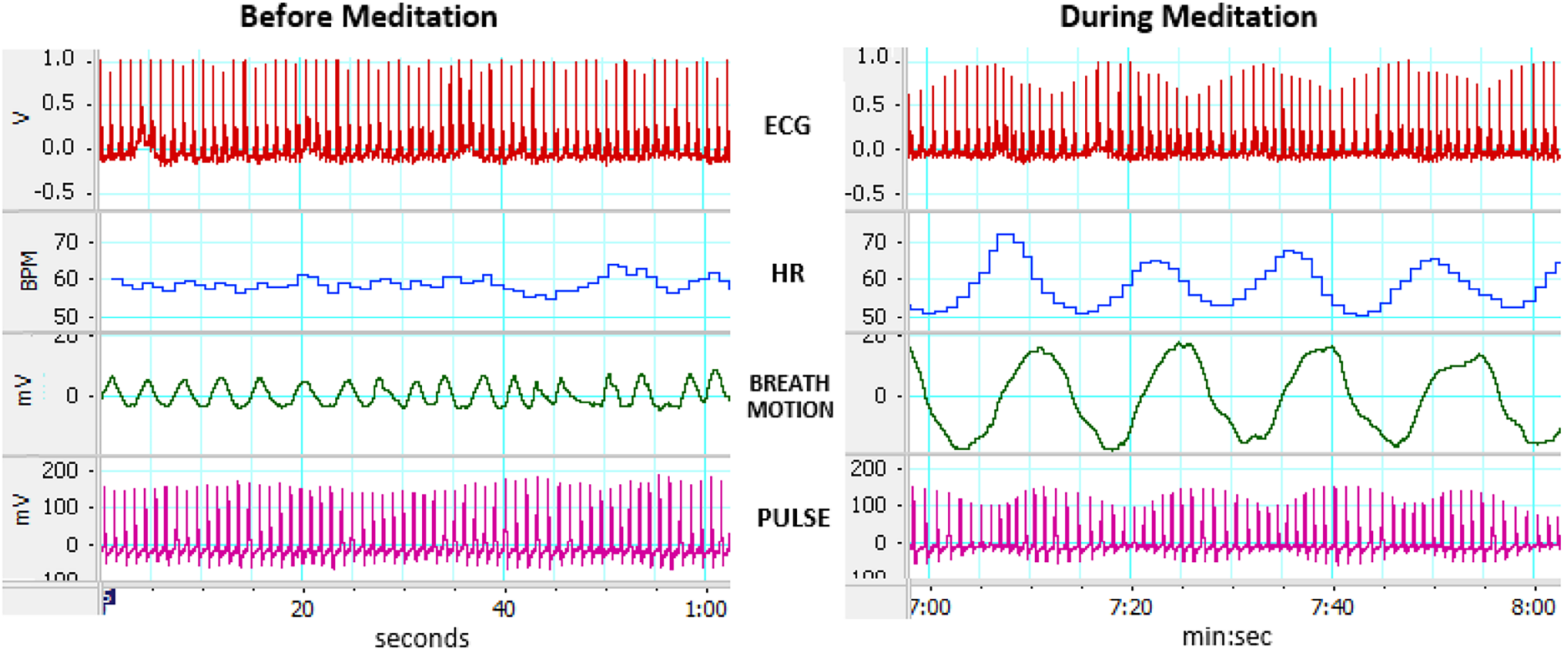
ECG, heartrate, breathing, and pulse before and after HRM

Slow rhythmic breathing produces oscillations in heart rate in response to stimulation of the vagus nerve through activation of two reflexes that are often referred to in HRVB studies. During inhalation stretch receptors in the lung increase firing rates in afferent fibers of the vagus which inhibits vagal outflow to the heart thereby increasing heart rate. During slow expiration vagal nerve activity is activated and heart rate is reduced (Chang et al., 2015; Eckberg, 1983). We show that the deep inhalations in HRM are associated with an initial fall in blood pressure (indicated by the pulse pressure signal in Fig. 1) then a rise in blood pressure. The rise in blood pressure activates the baroreflex. The baroreflex is a reflex mediated by blood pressure sensors in the aorta and carotid artery. The sensors are activated by stretching of the arteries as blood pressure increases. When blood pressure increases, the baroreflex causes a prompt decreases in heart rate (Lehrer & Gevirtz, 2014). Interestingly at resonance frequency breathing (about 5.5 bpm), heart rate and blood pressure oscillate completely out of phase, such that blood pressure begins to fall as soon as the heart rate rises and blood pressure rises as soon as the heart rate falls. (See Fig. 1). This supports the role of the baroreflex in high amplitude heart rate oscillations (Lehrer & Gevirtz, 2014; Vaschillo et al., 2002).

When these vagal effects cause the heart rate to slow many other organs including the brain are also influenced by the vagal nerve output. At the resonance frequency (RF) breathing rate these reflexes act additively to produce the maximum vagal effects and changes in heart rate. Indeed, HRVB uses resonance frequency breathing rates and its effect produces improvement in emotional regulation. When HRVB was practiced twice daily at home over about a 3 month period it produced increases in resting baroreflex gain (Lehrer et al., 2003, 2020a).

One of the challenges with HRVB discussed in a recent metanalysis is the variation in the breathing practice and type of biofeedback intervention used in the 143 studies reviewed (Lalanza et al., 2023). Some studies had participants breathe at a previously determined RF, whereas others made use of a biofeedback device to determine RF, while others simply had participants breathe at the rate of six breaths per minute. Further, the number of weeks practiced varied widely across studies. Nevertheless, the findings along with those of earlier studies and meta-analyses (Fisher & Lehrer, 2022; Goessl et al., 2017; Lehrer et al., 2020a) have found that participants practicing HRVB have shown numerous clinical benefits with the largest effect size on decreased depression and anxiety. Stress stimulates the sympathetic branch of the autonomic nervous system and is involved in the fight or flight response. The sympathetic nervous system accelerates the heartbeat while the parasympathetic system slows it. In these times of chronic stress, it is desirable to increase vagal tone: hence, the interest in self-management modalities that enhances it (Williams et al., 2015).

We propose that heart rhythm meditation (HRM), with its emphasis on the slow rhythmic full breath, is one of those modalities that can increase vagal tone and is a self-management strategy that makes use of the long full slow breath of 3.5 to 7 breaths per minute. Importantly, the rate of breathing achieved is a byproduct of adopting a breath duration of 6–8 heartbeats for both inhalation and exhalation respectively. This range covers the resonance frequency that produces the most pronounced vagal heart rate slowing found in the HRVB studies, and having participants breathe at this rate in this study is equivalent to what was done in some of the HRVB studies (Lalanza et al., 2023). Furthermore, vagal tone is directly proportional to the volume of the breath so one can expect breathing the full breath at 3.5-7 bpm would produce the strongest stimulation of the vagus nerve (Fisher & Lehrer, 2022; Lehrer et al., 2020a; Lehrer et al., 2020b). Figure 1 below shows the recording of the physiologic signals for a subject at rest and subsequently during HRM while breathing approximately 4.5 bpm. This recording was taken during preliminary studies, we haven’t measured breath motion or pulse during this study.

Figure 1: Physiological recordings of a subject immediately before meditation while reading from a book for one minute then during HRM. Recodings were done with an AD Powerlab of the Electrocardiogram (ECG), Heart Rate b/m (HR), Breath Motion from a sensor on the abdomen that measured the movement of the abdomen. Inspiration creates a positive deflection. The Pulse Amplitude measures the blood pressure and strength of the pulse. At rest, the heart rate changes with respiration are difficult to observe. However, during meditation the full breath produces larger amplitude changes in the breath motion sensor and is accompanied by clear oscillations in heart rate at the same frequency as breathing. Interestingly, the increase in heart rate is followed by a rise in pulse amplitude with a typical delay of about 5 s. The rise of pulse amplitude is indicative of the rise in blood pressure. The rise in blood pressure activates the Baroreflex to slow the heart (Lehrer et al., 2020a). The rise in blood pressure slows the heart at the same time as exhalation begins. This is an example of resonance between the RSA and the baroreflex.

While vagal tone is important, HRV as a measure of vagal tone is certainly not the only assessment of the value of a heart rhythm meditation practice. Many studies show that meditation of various types helps people manage stress and increase their well-being (Galante et al., 2016; McCraty, 2022; Pandya, 2021). Further, many meditation practitioners attest to how it has affected their overall quality of life, and studies of meditation and well-being support such effects (Sylapan et al., 2020). This connects to the notion of neurovisceral integration and emotion regulation.

### Neurovisceral Integration, Emotion Regulation and Well-Being

Thayer and Lane (2009) first described “a model of neurovisceral integration in which a set of neural structures involved in cognitive, affective, and autonomic regulation are related to heart rate variability (HRV) and cognitive performance (p. 141). This model suggests that the autonomic nervous system is controlled by cortical and subcortical brain regions, which controls the inhibition of what would otherwise be a stress response. HRV provides a window into assessing this inhibitory influence. More specifically, the model of neurovisceral integration proposes that vagally mediated heart rate variability (vmHRV) represents a psychophysiological index of inhibitory control of selective cortical structures like the prefrontal cortex over subcortical structures (e.g. the amygdala). These structures play a role in emotion regulation (Thayer & Lane, 2007; Williams et al., 2015), interoception, and sense of self (Kelley et al., 2002; Northoff et al., 2006)—functions that are central to the subjective experiences of HRM.

HRV can be regarded as an index of interaction between these higher brain structures, the autonomic nervous system and its effect on the physiology of the body, which affects emotion regulation, a process by which individuals modify their emotional experiences, expressions, and subsequent physiological responses based on their experiences (Aldao, 2013). Inhibitory control is a key mechanism for successful emotional regulation; individuals are required to inhibit what might be otherwise regarded as excessive emotional responses in service of more desirable and appropriate ones (Lane et al., 2009). When this inhibitory function is weak or absent the body responds as if stressed and anxious. Another way of viewing this situation is when the prefrontal cortex is hypoactive it fails to inhibit the amygdala (this is referred to as disinhibition) and the sympatho-excitatory circuits that are normally essential for energy mobilization during the “fight or fight” response. When this state is prolonged it produces the excess wear and tear on the body and brain and Lehrer et al. (2020a) have recently summarized studies that show the association of both the unmitigated interaction of aging and stress will decrease the cortical volume and thickness in areas associated with emotion regulation, and will affect both the brain and the heart (Lehrer et al., 2020a). Nevertheless, they also report the many studies and reviews that show how HRV mediated activity enhanced by interventions and increased coping mechanisms can increase cortical thickening in these areas and lead to greater emotion regulation and feelings of well-being (Lehrer et al., 2020a). Strategies that enhance the function of the prefrontal cortex serve to restore this inhibitory influence with improvements in mood and a sense of well-being. We suggest that HRM is one such strategy, particularly with its emphasis on the full breath.

## Methodology

We made use of a mixed methods research study design (Creswell & Creswell, 2018). Essentially, this is a sequential mixed methods study design, where the quantitative data were collected first to answer the first research question, related to how HRM affects vagal tone (by measuring changes in HRV before and after learning HRM and engaging in the meditation practice). We gathered both quantitative and qualitative data to answer the second research question related to how HRM affects well-being. The data collection methods will be discussed in detail below, after explaining our inclusion criteria, and how we ensured that they learned and would engage in the meditation.

### Sample Recruitment, Inclusion Criteria, and Informed Consent

We began recruiting participants in January of 2022. We aimed to gather a sample based on all of the following inclusion criteria:

- English speaking adults over the age of 18.
- Had to agree to participate in a 6-week course of instruction in HRM over Zoom.
- Had to agree to attend two in person meetings, where ECG measurements and well-being questionnaires were completed, and a zoom meeting where their experiences during practice were discussed.
- Had to agree to meditate every day for at least 20 min once the data collection began (after the third class in the course).

Exclusion criteria were that potential participants could not already be practicing HRM regularly (more than twice a week) prior to starting the study.

Once we received institutional review board approval, we advertised on digital media through the IRB’s Study Finder, as well as at a local medical center, local health studios and gyms, both to take the 6-week course, and to participate in the study. (Seventy-four participants enrolled in the study, but only 48 came for the post study appointment. The study participants signed the informed consent during the first in-person meeting.)

### Brief Course Overview

Participants were invited to participate in an online instructional program in HRM. This took place in weekly online zoom-based instruction sessions by one of our investigators who had eight years of experience teaching HRM at the Hershey Medical Center in Hershey PA. Approximately 200 registrants attended this course online that was provided at no charge to the participants. From these participants, we recruited 74 participants to enroll in the research study. Participants were required to come to the research laboratory at the Penn State College of Medicine in Hershey PA so participants were predominantly comprised of local residents.

Participants were taught the technique in incremental steps so that by the third weekly lecture we assumed that they were able to perform the technique independently. The meditation training included how to sit, how to breathe rhythmically, deeply and slowly by engaging the abdominal muscles, and synchronizing the breath with the heartbeat felt in the chest. The heartbeat served as a measure of the inhalation and exhalation length, approximately 6–8 beats on the inhale and the same length on the exhale (corresponding to a measured range of 3.5–7 bpm depending on one’s heart rate). The participants were also instructed in the cognitive and affective components of the technique. This included awareness of the physical mental and emotional experience. Implicit in the participation with this course was the intention to improve well-being through the practice of HRM and to improve emotional awareness and capacity. We felt that the conscious breathing practice would produce physiological changes that would be supportive of the desired experience of meditation. The course sessions set the expectation for the experience that might occur during meditation and prepared the participants for the practice.

### Quantitative Data Collection

Participants completed the six weekly lecture-instruction sessions, and were asked to meditate 20 min every day for 70 days (meditation period) and keep a digital log of the time spent meditating. Participants were invited but not required to attend coaching sessions that were held online by three of our team members weekly. These were made available to answer questions and encourage participation.

Study participants attended two in person meetings where ECG measurements were recorded and the well-being questionnaires were filled out. The first visit occurred before the third-class meeting session, when the formal meditation minutes collection started, and the second meeting occurred in the period of 4 weeks upon 70 days meditation practice period was over. During the first visit the consent was discussed and signed. The remaining protocol was identical during both meetings.

The ECG standard recording leads were applied to the skin of the arms and lower abdomen, and after the subject sat and rested for about 10 min, a 10 min-long ECG recording for HRV analysis was obtained. Both baseline and post-study recordings were done while the participants were resting and not meditating. During the second visit, the participants were additionally asked to meditate for 3–5 min while the ECG recording was taken. This recording during meditation was done to see if the heart rate of the subject oscillated in response to the slower and fuller breath that occurs during HRM. A clear oscillating pattern of heart rate changes in response to the slower breathing rate at a frequency of approximately 3.5–7 bpm was looked for and required for determination of adequate skill in HRM.

Immediately afterwards, participants filled out the two well-being inventories, chosen because they have been widely used and are validated instruments. These inventories are the WHO-5 and the Warwick-Edinburgh Mental Well-being Survey (WEMWBS). The WHO-5, developed by the World Health Organization in 1998, is a widely used validated index of five questions that measures “subjective well-being” or how people perceive their internal sense of well-being, as evaluated by a Likert scale (from 0 for not at all, to 5 which is all of the time). (See Appendix A, Fig. 5, for a list of the five questions). By contrast the WEMWBS developed in 2006, is a 14-item quantitative Likert-scale validated questionnaire used to assess mental well-being (thought and feelings) related to the prior 2 weeks (each item was stated positively and scored from 1 to 5, corresponding to none of the time to all of the time. The questions are similar to the WHO-5 but also include statements about optimism, relationships, and the person’s sense of being loved (See Appendix B, Fig. 6). The two inventories together provide a more holistic understanding of the person’s well-being. They were also asked to record the number of minutes they meditated each day into weekly electronic surveys created in a REDCap database or on a paper log sheet. In addition, they filled out a form indicating whether or not they had prior experiences with meditation, and were asked to identify which type.

### Quantitative Data Analysis

The ECG recordings were done with an AD Powerlab 16/30 and reviewed manually for artifacts using the AD Instruments LabChart Heart Rate Variability software module. A 10 min-long file containing the interbeat intervals without ectopic beats was analyzed for HRV using standard methods (Liao et al., 1995). We performed a spectral analysis of the frequencies extracted from the RR intervals and expressed the results in the form of the natural log (nL) as they were not normally distributed. From the power spectral density, two frequency-specific spectral powers were calculated, based on a rectangular method: Low Frequency (LF), defined as the power between 0.025 and 0.15 Hz band; and High Frequency (HF), the power between 0.16 and 0.35 Hz band. This frequency includes the normal breathing frequency for adults at rest (i.e. while not meditating). The ratio of LF/HF was also calculated. Time domain parameters including SDNN and RMSSD were also calculated as measures of improved heart rate variability and vagal tone respectively. The ECG recordings were adjusted for the time of recording to account for the natural time dependent rhythms of vagal tone. Key measures of increased vagal tone would be reflected in a rise in HF power, and a rise in RMSSD.

We considered that changes in HRV may depend on the amount of time spent doing the meditation practice. We anticipated that some participants would spend more time meditating than others and were interested in determining how the time spent meditating would influence the outcomes of interest, especially the HRV parameters. Accordingly, we did an adjusted linear regression analysis to look at the effect of time while we controlled for other factors which could potentially influence the outcome. These confounding factors included sex, age, baseline HRV, the time of day the measurements were made, whether the participants were doing any other exercise program, and baseline well-being scores.

The WHO-5 and WEMWBS survey instruments were reproduced in a REDCap database with copyright statements. The answers to each question were coded by corresponding number related to the number on the Likert scales, and later added to compute the final wellbeing scores. The results were analyzed in two ways, first, by using adjusted multivariable linear regression analysis controlling for age, sex and initial wellbeing scores (all participants included). Second, given that an increase in well-being scores was not necessarily linear with increase in meditation time, we also used a paired *t* test to see if there was a statistically significant increase in well-being scores for participants who meditated more regularly (at least 60% of days throughout the study period, and an average meditation time > 10 min/day, 42 participants). We looked separately at six participants who meditated less than 60% of the days primarily at the onset of the study and whose average meditation time < 10 min/day.

### Qualitative Data Collection and Analysis

While the quantitative data in the study are extremely important, it does not tell us what participants’ experiences were of doing heart rhythm meditation, or about their perceptions of how it affected their overall quality of life. Given that the purpose of qualitative research is to examine participants’ experiences and perceptions related to particular phenomena (Creswell & Creswell, 2018; Merriam & Tisdell, 2016), focus groups were conducted with several participants in the study who did the required meditation to examine their experiences of the phenomena. Given that some participants were not available at the times the focus groups were conducted, they were given the option of filling out a survey asking the same questions focusing on their experiences of HRM and how they believe it affected their overall quality of life, including their stress levels, relationships, work lives, and their experiences of love, harmony, and beauty. These focus groups were conducted by EJT of our study team, who has considerable expertise in qualitative research, and RB who assisted in the data analysis. A total of 18 participants engaged in the focus groups, and an additional 17 filled out the qualitative survey.

The research team made use of Nvivo (version 1.6.2), a qualitative data analysis program, to assist in the analysis. Two members of the research team (EJT and RB) engaged in an open coding process of data analysis; they then developed a code book, and a more in-depth coding of the qualitative data was done independently by these same members of the research team, while making use of Nvivo. The coders were in an agreement of the three primary themes overall, with a fourth theme that is relevant only to the more experienced meditators in the study.

## Quantitative Results

We present the quantitative results of the study, first by discussing the study participants and their meditation time and skill level to give these findings some context. Next we present the multivariable linear regression results of the HRV data and the well-being data, Finally, we discuss further results of the well-being data from the WHO-5 and the WEMWBS inventories,

### Study Participants’ Meditation Time and Skill

The participants were asked to practice HRM for 20 min every day during 10-week period, but not everyone practiced every day and some people practiced less than 20 min/day, therefore the average daily time (total time meditated divided by 70 days) varies from participant to participant.

**Fig. 2:**
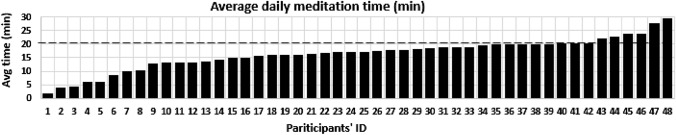
Time spent meditating

Figure 2 shows the average daily meditation time for each participant.

14 people meditated on average 20 min/day or more, but only five participants met or exceeded the study goal of meditating 20 min every day. There were six participants who meditated on average less than 10 min a day; they also meditated for less than 60% of the days. For clarity purposes, the participant’s ID was assigned based on the increasing order of the participants’ average meditation time.

Figure 2. The figure shows the participants’ average daily meditation times over the 10-week period (total minutes divided by 70 days). The study requirement was practicing 20 min/day, but not everyone practiced 20 min every day therefore the average daily time varies from participant to participant. Six participants meditated for less than 10 min a day and also meditated < 60% of the study days.

**Fig. 3:**
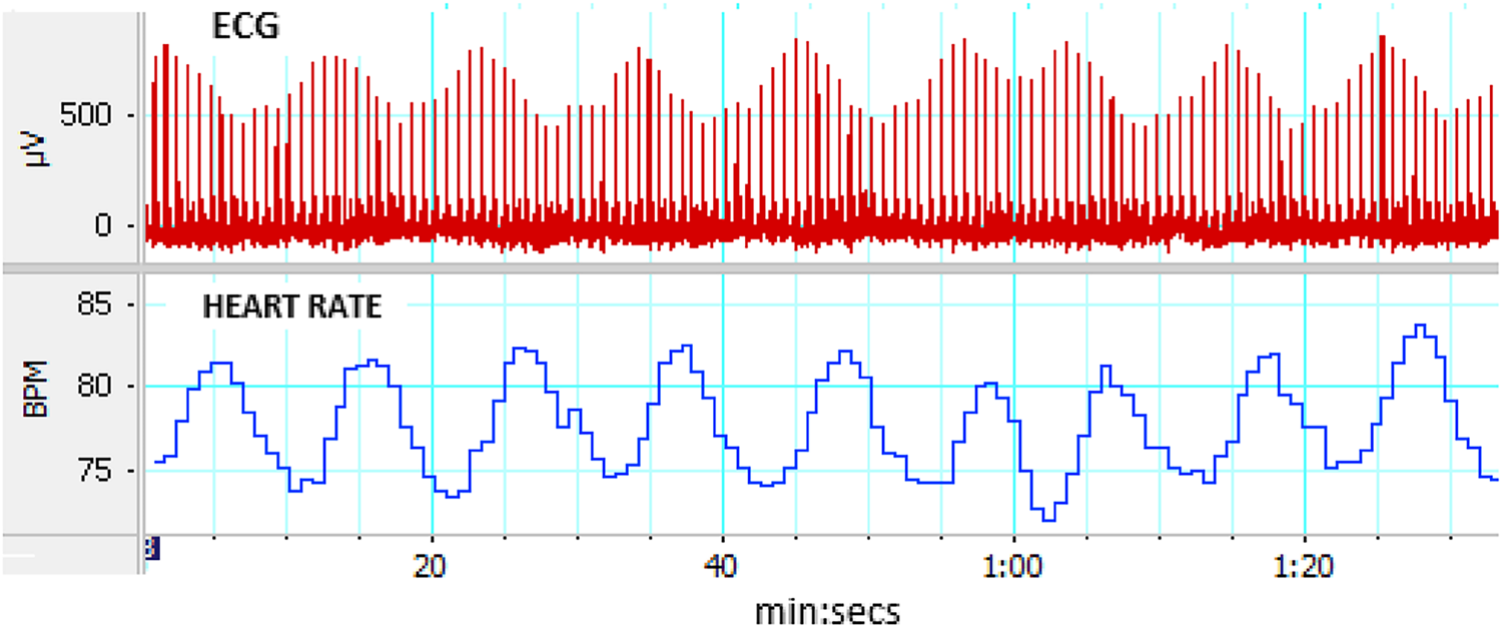
Example of participant with good skill

During the second study visit participants’ ECG and HR was recorded during resting time (for HRV evaluation) and then they were asked to meditate 3–5 min for evaluation of their HRM skills which we defined as the ability to generate a clearly visible oscillation in heart rate at the breathing frequency. Figure 3 below shows the oscillating ECG and HR signals of a participant who demonstrated ability to maintain slow full rhythmic breath (characteristic for HRM), which creates oscillations in the HR and ECG amplitude. 38/48 participants demonstrated adequate skills, while 10/48 participants did not demonstrate adequate skills.

Figure 3 An example of an ECG recording from a subject meditating during the second ECG recording after 10 weeks of practice. The ECG is shown above the heart rate tracing. A clear oscillating waveform of heart rate changes at about 6 cycles/min is evident. There are also amplitude changes in the ECG signal that oscillate with the same frequency as the heart rate changes. This is an example of waveforms where skill in meditation is present. 38/48 participants demonstrated adequate skills, while 10/48 participants did not demonstrate adequate skill.

### Results of Multivariable Linear Regression Analysis of HRV and Wellbeing Scores

The entire analytical sample for HRV consisted of 45 participants with a mean (SD) age of 56 (12) yrs. (Note of the 48 total number of participants 3 were excluded for HRV analysis because they had an excessive number of ectopic beats). Of the 45 participants, 33 (73%) were females.

Table 1 below shows the results of the multiple linear regression results. The table shows that for a 10 min increase in meditation time a day, RMSSD increases by 5.69 ms (or 0.569 for every 1 min meditation time per day). Although not significant an interesting trend was observed as all HRV parameters and well-being scores improved in a consistent manner. There was no consistent association between other factors (such as gender, and baseline well-being scores) and post-training HRV.

**Table 1:** Factors associated HRV after meditation and Well-being scores

Baseline Major factors	β (SE), p value of the “After” HRV and Wellbeing scores							
	Ln-HF	Ln-LF	LF/HF	SDNN	RMSSD	HR	WEMWBS	WHO-5
10 min/day meditation time	0.44 (0.32), p = .18	0.09 (0.27), p = .75	− 1.58 (1.96) p = .43	3.74 (5.32), p = .49	5.69 (4.09), p = .17	− 1.03 (2.59), p = .69	2.15 (1.96), p = .28	0.80 (1.13), p = .48
other exercises (Yes = 39)	0.23 (0.47), *p* = .63	− 0.43 (0.41), p = .30	− 3.54 (3.08), p = .26	− 8.81 (7.94), p = .27	0.44 (5.94), p = .94	− 3.38 (3.70), p = .37)	− 4.47 (3.06), p = .15	− 2.60 (1.80), *p* = .16
Baseline HRV	0.70 (0.19), *p* < .01	0.40 (0.13), p < .01	0.27 (0.27), p = .33	0.52 (0.23), p = .03	0.47 (0.22), p = .04	0.68 (0.15), p < .01		
Baseline WEMWBS index*	0.05 (0.03), p = .11	− 0.01 (0.02), p = .61	− 0.22 (0.18), p = .23	0.12 (0.47), p = .80	0.25 (0.35), p = .48	− 0.01 (0.22), p = .98	0.61(0.18), p < .01	
Baseline WHO-5*	0.05 (0.04), p = .21	− 0.02 (0.04), p = .58	− 0.27 (0.26), p = .30	− 0.29 (0.67), p = .68	0.05 (0.50), p = .92	0.16 (0.31), p = .60		0.61 (0.15), p < .01
Age	− 0.02 (0.02), p = .29	− 0.03 (0.01), p = .03	− 0.01 (0.09), p = .93	− 0.69 (0.30), p = .03	− 0.51 (0.21), p = .03	− 0.08 (0.11), p = .50	− 0.10 (0.09), p = .28	− 0.07 (0.06), p = .25
Sex (Female = 33)	− 0.03 (0.36), p = .93	− 0.21 (0.32), p = .51	− 0.24 (2.33), p = .92	− 1.61 (6.18), p = .80	− 1.19 (4.67), p = .80	3.73 (2.85), p = .20	1.11 (2.33), p = .64	2.06 (1.38), p = .15

Adjusted for timeframe of ECG measurements

All variables entered into the models together, except for *noted variable. Thus, the relationship between one variable has been adjusted for the potential confounding by other variables

∗These two well-being scores entered the models separately

### Further Results Related to the WHO-5 and WEMWBS Wellbeing Surveys

Given that the increase in well-being scores was not necessarily linear with increase in average meditation time, we decided to evaluate the effect of HRM on wellbeing scores using a paired *t* test by grouping all the participants who meditated at least 60% of the days (> 10 min/day) together. Additionally, we compared the mean pre and post well-being scores for 6 participant who meditated < 60% of the time (< 10 min /day) using paired *t* test for both the WHO-5 and the WEMWBS well-being surveys. The results for the WHO-5 are presented in Table 2 below.

**Table 2:** Paired *t* test for the WHO-5 and the WEMWBS

WHO-5 pre and post study scores					
Meditation frequency and average daily meditation time		Mean	STDEV	N	P
> 60% of the days > 10 min/day	Pre	14.5	4.8	42	< .001
	Post	17.3	4.3	42	
< 60% of the days < 10 min/day	Pre	14.2	3.2	6	0.8
	Post	15.5	5.0	6	

WEMWBS pre and post study scores					
> 60% of the days > 10 min/day	Pre	49.4	7.4	42	< .001
	Post	54.6	7.3	42	
< 60% of the days < 10 min/day	Pre	49.7	5.4	6	0.8
	Post	50.0	8.8	6	

The results of the paired *t* test analysis of participants who meditated at least 60% of the days and for an average of at least 10 min/day suggests a significant increase in well-being scores measured by both WHO-5 and WEMWBS instruments

On the contrary there was no increase in scores for those who meditated < 60% of the days, < 10 min/day

**Fig. 4:**
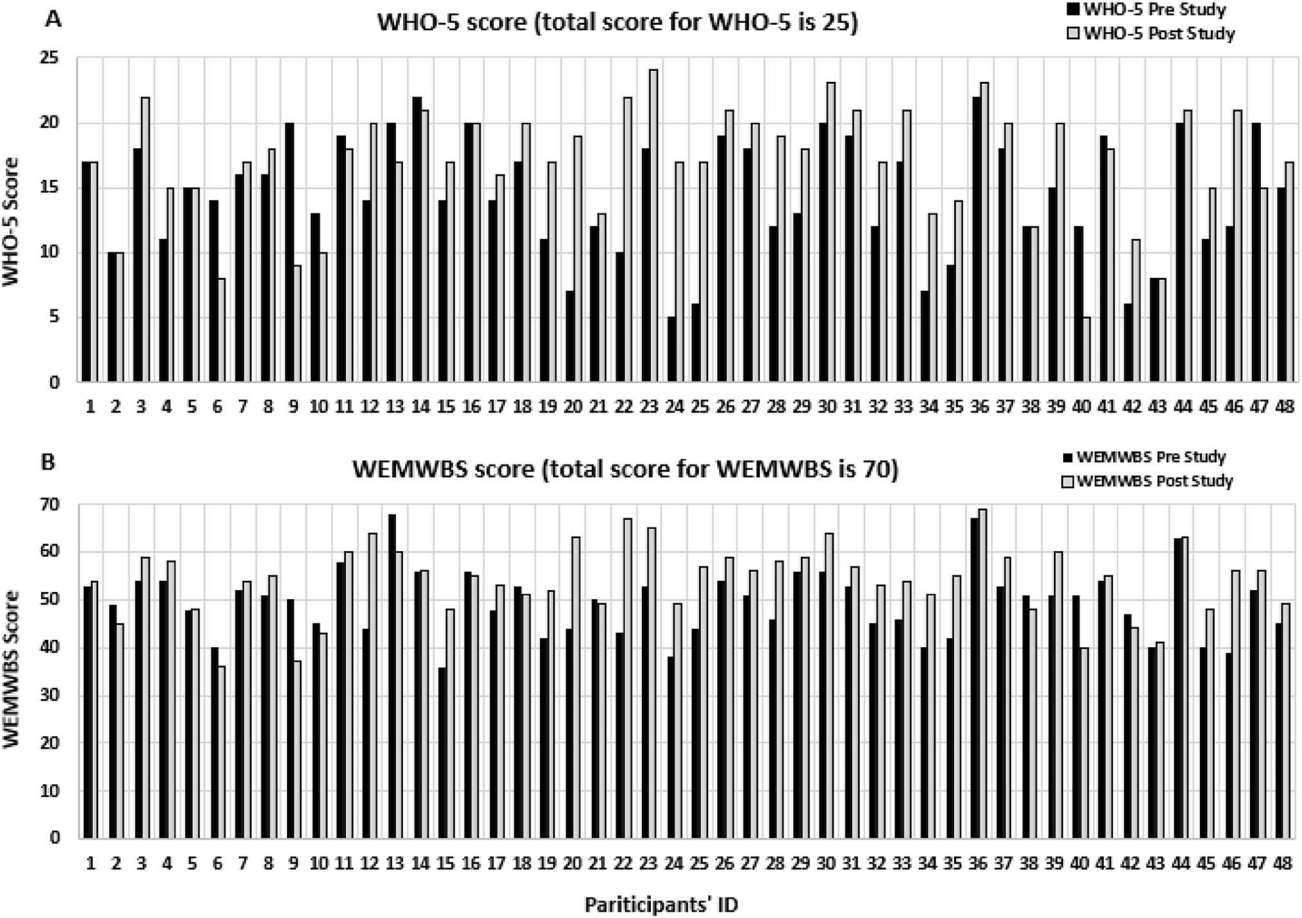
Shows individual pre and post WHO-5 and WEMWBS study scores for all 48 participants who completed the study

Figure 4 The upper graph (A): WHO-5 Pre and Post Study Score; The lower graph (B): WEMWBS Pre and Post Study Score for all 48 participants who completed the study. The maximum possible score is indicated on the graph for each instrument. Participants are arranged in the increasing order of their average meditation times reported in Fig. 2.

## Qualitative Results

A total of 35 of the 48 participants participated in the qualitative data collection of the study, by either participating in a focus group interview, or if they were unavailable during the focus group meeting times, by filling out a qualitative survey by answering the same questions that were asked in the focus group. While all 48 who completed the study were invited to participate in the qualitative portion of the study, only 35 did so. All of the participants indicated the value of the class that they took in HRM overall, and of hearing other people’s experiences of HRM and questions about it. An analysis of the qualitative data indicated three primary inter-related themes of findings related to all the participants in the study, and their perceived effects of HRM on their lives (summarized in Table 3):

1. The positive effects of HRM on stress and well-being.
2. The development of a more expanded sense of self.
3. An increased awareness of the interconnection of the body-heart-emotions.

**Table 3:** Example quotes from the three main qualitative themes

1. The positive effects on stress reduction & well being	
M. “It has given me renewed confidence in my value of just being present and helping calm down situations….”	
*A.Better Sleep*	F: It lets me be quiet and sleep well.
*B.* *Applying the Practice to Potential Stress of Daily Life*	M: It has given me renewed confidence in my value of just being present and helping calm down situations…. When I begin feeling pressured in traffic or other times of rushing, a few breaths help calm me down. HRM has increased my quality of life, not only for myself, but my time with friends and family.

2. A more expanded sense of self	
F: “I feel that *I am capable of much more than I imagined…*I think I have always had a keen sense of emotional awareness, but HRM has helped me work through some emotional situations more quickly.”	
A.*Increased self- esteem* B. *Greater awareness of Beauty*	M: I have become less judgmental about myself, and more able to differentiate between negative critical voices and helpful critical voices. F: It [HRM} helps me to feel better about myself. If I could boil it down it’s just like” you are not your job.”!! Like there’s a bigger self there, … there’s a self with a capital S
	F: it was after meditation. …I looked up the street and I live in the city and I looked up the street, and it was just so beautiful and I do not have her never seen it that way before : it was just beautiful.

3. Increased awareness of the interconnection of body-heart-emotions	
F: “HRM grounded me and made me more aware of my emotions and body sensations. Before HRM I regularly felt numb”	
	M: I love the practice of listening to my heart and pulling my center down to the heart. In those moments I begin to feel more whole and centered F: it's such a body full practice…When I focus on my heart and stay in my heart, you get a like a really very pleasurable sensation in my heart…it just feels like really, really good. and sometimes I’ll feel that sensation of extending to my other chakras so it's a very pleasurable physical sensation

### Summary and Implications of these Qualitative Findings

The first three themes of these findings—the positive effects on stress and wellbeing; an expanded sense of self; and an increased awareness of the interconnectedness of body, heart, and emotions—were essentially expressed in some way by all the participants who engaged in the HRM practice. The emerging fourth theme (summarized in Table 4 below) is related only to those participants in the study who had experiences with meditation prior to the study, though all the participants in the study were beginners of a regular daily practice of HRM. While further research would need to be done in this regard, this might suggest that deeper experiences happen in meditation over time with deeper and more sustained practice.

**Table 4:** Emerging theme for experienced meditators

4. (Emerging theme) more experienced meditators have deeper experiences	
a. *On being an antenna or a “channel”*	M. You know we’re sort of experience yourself like an antenna sometimes. …But you know it’s interesting the antenna notion, because sometimes like when you’re in the creative process = = whether I’ve been painting or writing a song–sometimes it feels like the song finds you or elements of the song come and you’re just channeling them for somewhere else …
b. *Connecting to prior contemplative experiences*	F: I have had several significant meditation experiences throughout the study. ***In early March, while I was meditating, I began to vibrate (at least that is what it felt like) - from the top of my head down to the tips of my fingers. It continued for another three minutes after my meditation ended. I had this experience on 2–3 other occasions,…My religious life is steeped in Ignatian spirituality and for over a decade I done an annual 7 day silent retreat. I am very much a pilgrim in the Ignatian way…

## Discussion

This was a mixed-methods research study that sought to examine how the practice of Heart Rhythm Meditation (HRM) affected vagal activity as examined by HRV, and how it affected participants’ well-being. Well-being was ascertained both by measuring changes in scores on two quantitative well-being inventories (the WHO-5 and the WEMWBS), and as participants discussed in qualitative focus groups about their meditation experiences or by answering these same questions in qualitative surveys. These sets of data together give a more holistic picture of the value of HRM (for those who actually did the practice), and the way that it affected participants overall lives. There are three discussion points to consider in order to make sense of the findings: contextual issues; the HRV findings; and the well-being findings.

### Contextualizing the Participation and Dropout Rates

Based on the data overall, it is clear that there are benefits to developing a sustained HRM practice. That said, there are three primary aspects to keep in mind when thinking about the study related to the participation and attrition levels. First, meditation studies overall often have a significant attrition rate for various reasons (Nam & Toneatto, 2016; Russ et al., 2017). Our study was no exception in this regard; roughly 1/3 of our participants appeared to drop out of the study, likely because they did not continue to meditate, at least with regularity. The study was designed to determine if a regular practice of HRM as learned remotely over zoom would affect vagal tone and well-being after 10 weeks of practice. As such the outcomes are dependent on participants practicing meditation on a regular basis.

Second, it is also important to point out that only 14 of the 48 participants who came for the second measure actually meditated for 20 min per day. This may suggest that establishing and maintaining a practice is more difficult or complex than one might think in its initial stages, when one initially thought that participating in a meditation study is a good idea. Clearly, one needs to carve out the time and space to do so.

Third, and related to the above two issues, is the fact that from the research literature about a variety of meditation studies and practices and the difficulty with attrition, we were aware that participants would likely need support in maintaining their practice. Our prior study also indicated the importance of having a community of support (a regular group to meditate with), once the instructional course is over to sustain the practice. This is why we established weekly synchronous online coaching sessions once the course was completed. While others in the HRM course took advantage of the coaching groups, for the most part they were not actual study participants. Our prior studies also indicate that regular meditators appreciate the value of having a mentor or coach to guide one through the variety of inner experience that meditation can uncover. For instance, beginning meditators may be put off by meditations that bring up memories that are uncomfortable unless understood within a psychospiritual development context (Russ et al., 2017). In any case, in reading the results of the study, it is important to keep in mind that while those who did the meditation practice did have significant positive effects, the fact that meditation studies (including this one) have attrition issues, may indicate that there’s some resistance to various meditation practices (including this one) that might be related to time, emotional discomfort that comes up, or a myriad of other factors that need to be kept in mind when making sense of the results of the study.

### The HRV Effects Approach Statistical Significance

The outcome of the HRV data is reported in Table 1. The results from the linear regression models suggests that increased average daily mediation time is associated with higher after-training HRV (vagal tone), lower heart rate and higher well-being scores. The direction of this pattern was very consistent across all HRV measures of Vagal Tone (including the key measures of Ln-HF, SDNN, RMSSD) and well-being scores. However, as mentioned above, due to the small sample size, or relatively short duration of the study (10 weeks) these patterns of associations did not reach statistical significance. But this can also be related to the issues of skill level related to creating oscillations in heart rate through the breathing practice, and the time actually spent meditating.

The HRV results are probably most sensitive to the time spent meditating and the ability to generate an oscillating heart rate pattern with breathing. Ten of the 48 participants did not show this pattern, and as such one can speculate that their meditations may have lacked the stimulation of the vagus through breath. HRM is a meditation technique that uses slow rhythmic breathing using the full breath. When performed effectively breathing produces oscillation changes in heart rate that are easily visible on a heart rate tracing (see Fig. 3). While most participants were able to demonstrate this heart rate effect there were 10 participants who did not clearly show this effect. One can only presume that they did not incorporate the breathing component of the technique effectively. The inability to demonstrate these heart rate changes suggests either that the participants had very low vagal tone to start with, or more likely, that they did not breathe fully and rhythmically as required in this meditation method. This is where having some HRVB device that gives them immediate biofeedback may be helpful, though the use of such devices is often not practical for the average meditator. The lack of heart rate changes in these participants suggest that the meditation did not stimulate vagal slowing of the heart. Thus, one would expect that their ability to induce lasting changes in vagal tone to be limited. These participants may well have practiced meditation without the effect of the full breath for many of the ten weeks, without realizing they were not incorporating the full breath. Perhaps if they had been aware of their heart rate changes they could have improved their technique. In future courses of meditation instruction, it seems ideal to provide participants with the biofeedback required so they may improve their technique. Technology for monitoring heart rate in real time is now available for the smartphone.

### The Importance of Time

The second potential limitation is the time spent practicing meditation. Participants were asked to meditate for 20 min a day for the full 70 days of the study. Only 14/48 participants meditated the required 20 min a day while 6 meditated less than 60% of the days (< 10 min a day on average). The remaining participants meditated on average between 15 and 20 min a day. Those who did between 15 and 20 min per day did have positive effects overall (as discussed below in the well-being section).

## Conclusions Related to HRV

It is encouraging that all the relevant parameters of HRV moved consistently in support of increased vagal tone (engagement of the parasympathetic) and less sympathetic tone. Given what we now know about HRV and how efforts to increase HRV have reciprocal neuroplastic benefits (Cho et al., 2023), there is likely to be increasing interest in validating approaches for doing so. HRM seems a likely candidate as it combines a number of synergistic mechanisms including lowering sympathetic tone (our preliminary studies have shown a reduction in skin conductance during HRM (Palmer et al., 2016) and an increase in vagal tone through slow rhythmic breathing. The emphasis on developing positive emotion and emotional capacity is also an effective strategy (Tiller et al., 1996) and a key outcome and goal of the full spectrum of HRM practices. Interestingly, research has shown that intranasally administered oxytocin (the cuddle hormone) also increases HRV (Kemp et al., 2012).

### Improvement in Well-being

The purpose of the study was not only to examine the vagal effects of doing HRM, but it was also to examine how the practice affected participants’ well-being. Participants overall improved in well-being, which was the second major research question in the study. Below we discuss the quantitative results, then the qualitative results.

### Quantitative Results Show Significance

**Fig. 5:**
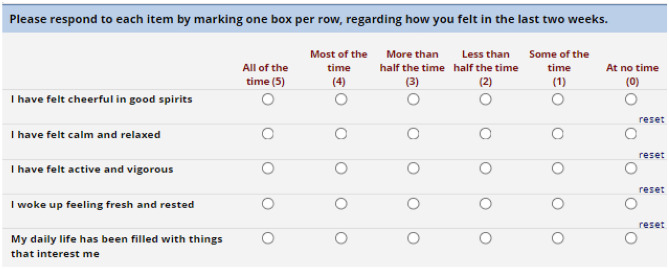
WHO-5 Well-being Index

**Fig. 6:**
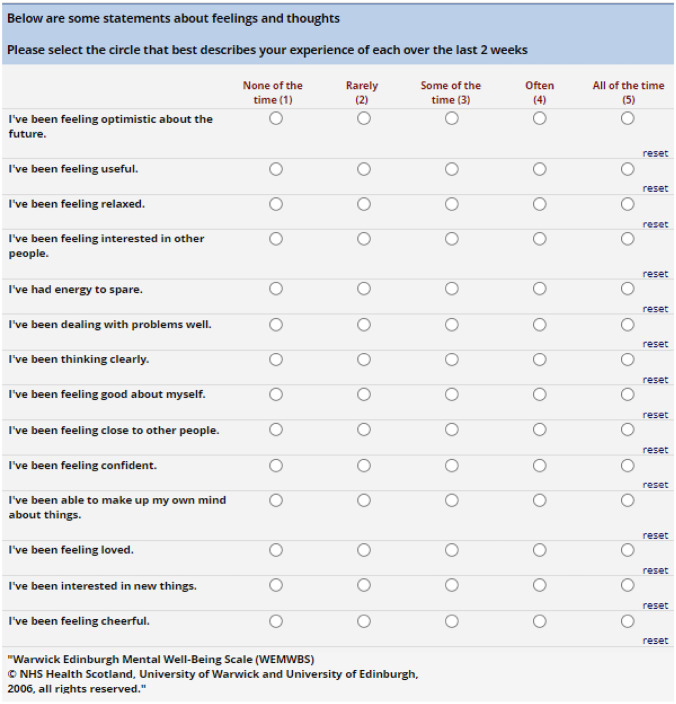
Warwick Edinburgh mental Well-being scale

Both of the well-being surveys (the WHO-5 and the WEMWBS) showed a modest increase in average well-being scores for the group of participants who meditated > 60% of the days (and on average > 10 min/day over 70 days study) that were statistically significant (See Figs. 4 and 5; Table 2). There were six participants who completed the study who meditated less than 60% of the days mostly in the first part of the study (or on average < 10 min per day). There was no improvement in their average well-being score (See Table 2).

The regression analysis showed that the most significant predictor of post study well-being scores were initial well-being scores, meaning the higher the initial scores the higher the post-study well-being scores. However, it is worth mentioning that a number of participants whose initial scores were on a lower side who meditated regularly had quite significant improvements. Specifically, 4 participants increased their WHO-5 scores by 11–12 points, and the same individuals increased their WEMWBS scores by 11–24 points. That is more than two points per question on the WHO-5 questionnaire (comprised of 5 questions) and approximately 1-point improvement per question on the WEMWBS instrument (comprised of 14 questions). Since both WHO-5 and WEMWBS questionnaires comprise of positive statements, this means that participants’ subjective estimate of the time “felt cheerful and in good spirits”, for example, might have increased from “some of the time” to “more than half of the time” or from “less than half the time” to “most of the time”. This level of improvement across entire questionnaire suggests that individuals with low initial scores can significantly improve their well-being scores if they meditate regularly. It is also worth mentioning that some participants whose well-being scores did not improve stated during the second in-person meeting that regular meditation practice helped them throughout the very stressful times they were experiencing at the time.

While most participants improved their well-being scores overall, the increase in wellbeing scores resulted in pattern of association with increase in meditation time but this pattern was not statistically significant at the traditional *p* < 0.05 level. Given that a variety of other external factors might have also affected participants’ well-being scores in the two weeks prior to taking the survey, and a small sample size, the results of regression analysis are still encouraging even though the statistical significance was not observed.

For those who did meditate regularly, the positive effect on well-being is in keeping with other breathing practices discussed earlier and is likely to be related to a combination of effects directly related to the practice of HRM. These include a reduction of stress, and an increase in parasympathetic activity. With these changes we can expect an improvement in emotional regulation and in awareness that comes with the central benefits of improving vagal tone.

### Qualitative Results Show Highly Positive Effects of the Practice

Due to space limitations, we only provided here the qualitative results in the form of a table. Nevertheless, the qualitative results did indicate that there were significant increases in overall well-being from doing the meditation based on what participants said about their HRM meditation experiences, and its perceived effects. It is important to note here that we only included people in the focus groups that actually did the meditation, as the purpose of the study was to examine the effects of the meditation itself on participants’ lives overall from their own perspective. There was no point in asking this of people who did not do the meditation.

Of those interviewed or who provided written answers to the focus group questions, there were three main inter-related findings related to all the participant responses:

1. the positive effects of HRM on stress and well-being.
2. the development of a more expanded sense of self.
3. an increased awareness of the interconnection of the body-heart-emotions.

Participants’ comments as reported in the qualitative findings section of this report, were quite striking about the ways the practice was benefitting them. Perhaps most striking was not so much the benefits to how they managed and were able to reduce stress in the moment, but rather their expanded sense of self that they experienced as a result of doing the practice. This likely comes from not only an awareness of their heart and breath and heart interaction, but more from an increased in understanding of their metaphorical heart, in comments like:

*I feel that* ***I am capable of much more than I imagined****…I think I have always had a keen sense of emotional awareness, but HRM has helped me work through some emotional situations more quickly.*

This expanded sense of self also relates directly to the third main theme regarding the increase in the awareness of the inter-connection of the body, heart, and emotions, that is also evident in the above quote.

Interestingly, there is an emerging fourth theme that is relevant to only the more experienced meditators in the study, and the fact that they had much deeper meditation practices that appear to be somewhat spiritual in nature. This likely suggests that the longer one regularly meditates (in terms of months, and years) the more likely one will have deeper experiences overall. Perhaps it is the neuroplastic changes in the brain that results in a greater understanding of a more expanded sense of self that can lead to deeper experiences.

### Well-Being Conclusions

The findings of the quantitative and the qualitative together indicate the positive effects on well-being and quality of life. The benefits are evident in the increase in well-being scores on both the WHO-5, and in the WEMWBS in the vast majority of participants who practiced meditation more regularly—at least 60% of the days for an average of at least 10 min per day as indicated in Figs. 4 and 5; Table 2. Further, the qualitative findings support the fact that such participants report a decrease in their stress level, an increase in their quality of life, and a more expanded sense of self that has increased their quality of life. This increase in well-being and quality of life overall indicates the benefit of HRM.

## Conclusions and Recommendations for Further Research

In summary, this study suggests that HRM is a powerful method for generating high amplitude oscillations in heart rate that contribute to increase in HRV. Most of the participants who practiced HRM regularly (> 60% of the time) for at least 10 min a day during the 70-day study, had increased well-being scores as measured by WHO-5 and WEMWBS well-being instruments. In addition, multiple HRV measures improved with time spent meditating, suggesting improved vagal tone. Considering the association between baseline HRV and the after mediation HRV and that the individuals who spent more time in meditation tend to have a better baseline and post medication HRV, we expect that they shall benefit more in terms of the autonomic balance from the meditation in a longer time if they continue to do so beyond the study period reported in our current study.

There is rising interest in the research literature on the value of strategies for improving autonomic regulation by increasing vagal tone. The primary strategy currently being used by investigators is slow paced breathing at approximately 0.1 Hz using heart rate variability biofeedback to produce large oscillations in heart rate. The benefits of these practices are just being appreciated and include improvements in anxiety, and mood and even a reduction in plasma proteins associated with Alzheimer’s disease (Min et al., 2023). This study shows how HRM can be considered an additional strategy for producing oscillations in heart rate and thereby similar benefits on improving autonomic regulation and all its downstream benefits on health and preventing the aging process. As non-pharmacologic approaches to health and well-being are increasingly attractive, HRM has the potential to add to this short list of effective strategies. Moreover, it combines the physiologic benefits of enhancing vagal tone with the cognitive and affective benefits of the meditation practice that seems to also enhance emotion regulation. Further research in using HRM to explore the benefits of improving HRV by increasing vagal tone during the practice, should confirm that participants achieve oscillations in heart rate and support participant compliance with doing the practice. Incremental increases in meditation time and regularity of the practice should also be considered.

In conclusion, the study contributes to the emerging research base that has captured the interest of scientists and others in the past few years on the effects of contemplative and other practices on vagal activity (as measured by HRV), and well-being. It provides evidence that HRM with its emphasis on the heart and full breath can be counted as one of the effective contemplative practices that can enhance one’s vagal tone, well-being, and overall quality of life.

## Data Availability

No datasets were generated or analysed during the current study.

## Appendix

See Appendix Figures 5 and 6
